# Cardiometabolic risk pathways in adults with disabilities: A structural equation model of diet quality and functional ability

**DOI:** 10.1371/journal.pone.0354600

**Published:** 2026-07-23

**Authors:** Radwan Qasrawi, Suliman Thwaib, Ghada Issa, Rand Al Taweel, Razan Abu Ghoush, Malak Amro, Hazem Agha, Diala Abu Al-Halawa, Haneen Al Taweel, Raghad Amro, Ayoub Jawaldeh

**Affiliations:** 1 Department of Computer Science, Al-Quds University, Jerusalem, Palestine; 2 Department of Computer Engineering, Istinye University, Istanbul, Turkey; 3 The Center of Technology and Innovation, Al-Quds University, Jerusalem, Palestine; 4 School of Public Health, Al-Quds University, Jerusalem, Palestine; 5 Department of Medicine, Al-Quds University, Jerusalem, Palestine; 6 Regional Office for the Eastern Mediterranean, World Health Organization, Cairo Governorate, Egypt; University of Diyala College of Medicine, IRAQ

## Abstract

**Background:**

Adults with disabilities experience increased cardiometabolic risk, yet the pathways linking nutrition, functional ability, and environmental factors remain insufficiently defined. This study examined these relationships using a structural equation modeling framework.

**Methods:**

A cross-sectional study was conducted among 123 adults with disabilities in Palestine (mean age 31.9 years). Data included anthropometric measures, two non‑consecutive 24-hour dietary recalls, disability severity (Disability Rating Scale), functional ability (Barthel Index), psychosocial environment, and socioeconomic indicators. Dietary quality and adequacy were assessed using the Diet Quality Index (DQI) and Mean Adequacy Ratio (MAR). Seven latent constructs were specified and tested using confirmatory factor analysis and structural equation modeling.

**Results:**

Indicators of cardiometabolic risk were prevalent: 58.5% overweight/obese and 51.2% had inadequate intake. Disability severity correlated with reduced muscle mass and poorer diet quality. Nutritional adequacy positively predicted body composition (β = 0.274) and functional ability (β = 0.185). Functional ability and physical activity were inversely associated with adiposity, while sedentary behavior moderated the relationship between diet quality and waist circumference. Mental illness specifically impaired diet quality, but not caloric intake. Urban residence and lower education predicted poorer dietary outcomes. The model explained 63.7% of variance in body composition and 94.4% in functional ability.

**Conclusions:**

Cardiometabolic risk in adults with disabilities is shaped by interrelated pathways involving disability severity, nutrition, functional capacity, and psychosocial factors. Lower anthropometric measures may reflect muscle loss rather than reduced risk. Interventions should focus on improving nutritional adequacy, supporting functional independence, addressing mental health, and reducing environmental barriers.

## 1. Introduction

Cardiometabolic diseases, including cardiovascular disease, metabolic syndrome, and type 2 diabetes, are leading contributors to global morbidity and mortality [1]. Individuals with disabilities consistently reported higher prevalence of obesity, hypertension, diabetes, and related risk factors compared with the general population [2,3]. These disparities are not exclusively attributable to biological conditions but are strongly shaped by structural and environmental constraints, including limited access to healthcare services, reduced opportunities for physical activity, and barriers to healthy food environments [4]. Evidence from population-based studies indicates that adults with disabilities are more likely to engage in sedentary behaviors, experience unmet healthcare needs, and encounter socioeconomic disadvantages that cumulatively contribute to elevated cardiometabolic risk [5,6]. Collectively, these patterns underscore the need to examine cardiometabolic risk within a framework that accounts for functional limitations alongside behavioral and metabolic determinants.

The etiology of cardiometabolic disease in the disabled population is characterized by multiple interrelated pathways that extend beyond traditional risk factors. Functional limitations, restricted mobility, and environmental barriers influence both lifestyle behaviors and physiological processes, creating conditions that increase susceptibility to metabolic dysregulation. Individuals with disabilities often face challenges in maintaining regular physical activity and accessing preventive healthcare, which contributes to the accumulation of cardiometabolic risk over time [7,8]. In addition, disparities in nutrition, health literacy, and social support further shape exposure to risk factors and influence disease progression [9,10]. These consistent influences indicate that cardiometabolic risk in adults with disabilities should be understood as the result of interlinked behavioral, functional, and metabolic processes rather than isolated exposures. Analytical approaches such as structural equation modeling (SEM) are therefore appropriate, as they allow simultaneous estimation of direct and indirect relationships while accounting for latent constructs derived from observed variables [9,11].

Within this framework, cardiometabolic risk can be specified as a latent construct reflected by related biomarkers such as body mass index, blood pressure, lipid profiles, and glycemic indicators. These measures do not operate independently but represent interrelated metabolic processes that collectively contribute to disease development [12]. Modeling cardiometabolic risk as a latent variable provides a more accurate representation of its structure and allows examination of pathways through which upstream determinants influence health outcomes. In the present study, cardiometabolic risk is operationalized via anthropometric indicators of adiposity, which are strongly associated with metabolic dysfunction and are clinically meaningful proxies in this population.

Diet quality is a key determinant within these pathways. Literature has shifted from a focus on individual nutrients toward dietary patterns that reflect overall intake [1,13,14]. Diets characterized by higher consumption of fruits, vegetables, whole grains, and unsaturated fats, and lower intake of refined carbohydrates and ultra-processed foods, are associated with improved metabolic profiles and reduced cardiometabolic risk [15,16]. Evidence from SEM-based analyses indicates that diet quality contributes to cardiometabolic outcomes through both direct pathways and indirect pathways involving intermediate variables such as adiposity and metabolic dysfunction [12,15]. Moreover, in populations with disabilities, diet quality is shaped by additional constraints. Limited mobility, reduced access to transportation, and financial barriers can restrict access to healthy foods, while functional impairment may reduce the ability to prepare meals, leading to reliance on energy-dense and nutritionally poor options [8]. Studies have shown that individuals with disabilities are more likely to have lower diet quality compared with those without disabilities, which may contribute to their higher cardiometabolic risk [17–19]. These findings indicate that diet quality is both a determinant of cardiometabolic health and a factor influenced by functional and environmental conditions.

Functional ability, encompassing mobility, strength, endurance, and the capacity to perform activities of daily living, is closely linked to cardiometabolic outcomes and related health behaviors [20]. Reduced functional capacity is associated with lower levels of physical activity and increased sedentary behavior, both of which are independently linked to adverse metabolic and cardiovascular outcomes [20–22]. Functional limitations may also contribute to changes in body composition, including increased adiposity and reduced muscle mass, which are known risk factors for cardiometabolic disease.

Poor dietary intake contributes to muscle loss, inflammation, and reduced physical performance, which may accelerate functional decline. Conversely, functional limitations can constrain dietary choices and reduce the ability to maintain healthy eating patterns. Interventional studies have demonstrated that improvements in diet quality are associated with better physical functioning and quality of life, indicating that nutritional factors influence functional health [23,24]. Research studies have found that functional ability may mediate the relationship between diet quality and cardiometabolic risk, as dietary patterns influence metabolic status and physical function, which in turn affect health outcomes [9,25]. This pathway may be more important in adults with disabilities, where functional limitations and environmental constraints interact. In this context, cardiometabolic risk arises from multiple interrelated pathways involving diet, physical activity, and metabolic processes. Although SEM has been applied to capture these relationships by estimating both direct and indirect effects within a single framework [9,12,25], existing studies often examine diet quality and functional ability separately, with limited focus on adults with disabilities and limited use of models that account for mediation and latent constructs.

To address these gaps, this study applies an SEM framework to examine cardiometabolic risk pathways in adults with disabilities. Cardiometabolic risk is specified as a latent outcome, while diet quality and functional ability are modeled as key determinants. The analysis evaluates the direct association between diet quality and cardiometabolic risk, the independent contribution of functional ability, and the extent to which functional ability mediates the relationship between dietary patterns and cardiometabolic outcomes. This approach enabled the examination of interrelated pathways and contributes to understanding cardiometabolic risk in populations with disabilities.

## 2. Methods and materials

### 2.1 Study design

This cross-sectional study was conducted among adults with physical and/or cognitive disabilities in Palestine. A total of 123 adults aged 19–61 years were recruited from disability care institutions and community settings in 2024. Eligibility criteria included a confirmed diagnosis of physical, intellectual, sensory, or mixed disability, age ≥ 19 years, and ability to provide or have a guardian provide informed consent. Participants residing in camps (n = 4), villages (n = 40), and urban areas (n = 79) were included. Of the 123 participants, 91 (74.0%) resided in registered disability care institutions, while 32 (26.0%) lived with their families.

Participants were excluded from the study if they had an acute illness at the time of data collection, exhibited edema or medical instability that could affect the accuracy of anthropometric measurements, or had incomplete dietary or socioeconomic data.

The study protocol was reviewed and approved by the Institutional Research Ethics Committee of Al-Quds University (Ref No: 301/REC/2023). Written informed consent was obtained from participants or, when not possible, from their parents, guardians, or primary caregivers. Participation was voluntary, and confidentiality of personal information was maintained throughout the research process. Data was collected over a twelve-month period, from June 1^st^, 2024 to May 30^th^, 2025. The study design and reporting followed the STROBE guidelines for observational research.

### 2.2 Measures

#### 2.2.1 Disability measure.

Disability severity was assessed using the Disability Rating Scale (DRS) [26], a validated instrument covering eight subscales: eye opening, motor response, communication ability, feeding, toileting, grooming, performance level, and employability/adaptability. The DRS total score ranges from 0 (no disability) to 29 (extreme vegetative state), with higher scores indicating greater severity. Participants were also categorized using DRS severity classifications: Normal, Mild Disability, Moderate Disability, Moderate to Severe, Severe Disability, and Vegetative State. Etiology of disability was categorized into four groups: Congenital/Genetic, Perinatal/Birth-related, Acquired Medical/Infectious, and Trauma/Injury/External.

Functional ability was assessed using the Barthel Index, a 10-item measure of activities of daily living, including feeding, mobility, grooming, dressing, bathing, toileting, transfers, and continence [27]. Each item is scored based on the level of assistance required, with higher scores indicating greater independence. The scores were categorized into three outcome levels: total dependent, partial dependent, and total independent, reflecting increasing levels of functional ability. These items formed a reliable latent Function construct (ω = 0.906, AVE = 0.660) in confirmatory factor analysis. Two subscales, eye opening and motor response, were excluded from the latent construct due to poor inter-item correlations and sign inconsistencies with the core items.

#### 2.2.2 Anthropometric and body composition measures.

Body composition was assessed through four anthropometric measurements. Body mass index (BMI, kg/m²) was calculated from measured weight (kg) and height (cm) using standard protocols. Waist circumference (cm) was measured at the midpoint between the lower costal margin and the iliac crest, with metabolic risk defined as waist >80 cm in women and >94 cm in men (IDF, 2006) [28]. Arm circumference (cm) was measured at the midpoint of the upper arm and served as a proxy for muscle mass and lean body composition. Weight was recorded using a calibrated scale to the nearest 0.1 kg. A composite Body Composition latent variable was constructed in confirmatory factor analysis using all four indicators (see Section 3.4).

#### 2.2.3 Nutritional assessment.

Dietary intake was evaluated through two non-consecutive 24-hour dietary recalls (one on a weekday and one on a weekend), using the multiple-pass method to improve accuracy and reduce reporting bias. Trained nutritionists administered the recalls. Total caloric intake, macronutrient composition (protein, carbohydrates, total fat), and key micronutrients (iron, vitamin D, calcium, vitamin B12, vitamin A, folate, vitamin C, sodium) were computed using a standardized Palestinian food composition database. A Mean Adequacy Ratio (MAR) was computed as the mean of nutrient adequacy ratios for 24 micronutrients capped at 1.0, with MAR ≥ 0.7 classified as adequate dietary intake [29]. A Diet Quality Index (DQI) was computed as the sum of healthy food group frequency scores (fresh fruit, vegetables, dairy, eggs, fish) minus the sum of unhealthy food frequency scores (sweets, fast food, fried food, processed meat), ranging from −1–8, with higher values indicating better diet quality. Food frequency was assessed using an ordinal scale (≤ once/week = 1, 2–4 days/week = 2, ≥ 5 days/week = 3).

#### 2.2.4 Psychosocial environment.

The psychosocial environment was assessed using a structured questionnaire adapted from validated scales measuring family functioning, perceived social support, and psychosocial adversity. The questionnaire drew on items from validated measures of family functioning and psychosocial adversity (originally developed for general populations; adapted here for adults with disabilities) [30,31]. Two latent constructs were derived. Positive Care (Psycho) was measured by five items: someone cared for the participant, the participant felt important, felt loved, family members cooperated to help, family took participant to the doctor, and family encouraged participation (all rated: Wrong = 0, Correct = 1, Very True = 2) [30]. Psychosocial Adversity (Adversity) was measured by four items: being called lazy, perceived family rejection, feeling insulted, and frequency of harassment, with all item’s direction-corrected so that higher scores indicate greater adversity [31]. A psychosocial net composite (psych_net) was also calculated by subtracting the Psychosocial Adversity score from the Positive Care score, yielding a single difference score that reflects the balance between positive and negative psychosocial experiences. This composite was used in observed‑variable models that do not rely on latent constructs, allowing for complementary analyses alongside the structural equation modeling approach.

#### 2.2.5 Socioeconomic status and social participation.

Socioeconomic status (SES) was assessed using four indicators: car ownership (0 = none, 1 = one, 2 = two or more), computer/device ownership, guardian educational level, and room ownership. These formed a latent SES construct in the CFA. Employment status (working = 1, not working = 0), guardian employment status, the subject's own educational level (1 = less than secondary, 2 = secondary, 3 = higher), and residence type (1 = village, 2 = camp, 3 = city) were treated as observed variables in structural models. Institution membership (91 in care facility vs. 32 living with family) and institutional meals provision were also recorded.

#### 2.2.6 Physical activity and sedentary behavior.

Physical activity was assessed by asking participants whether they engaged in light, moderate, and heavy physical activity (yes/no). Sedentary behavior was measured as daily sitting time in hours, with values capped at 16 hours after reviewing outliers. To align with the latent Activity construct such that higher scores reflect greater physical activity, we reversed the sitting variable (sitting_rev = 16 − sitting). In addition, a metabolic equivalent of task (MET)‑weighted composite was calculated to summarize overall physical activity intensity using the formula: heavy × 8 + moderate × 4 + light × 2.

#### 2.2.7 Mental health comorbidity.

Co-occurring mental illness was recorded as a binary variable (Yes/No) based on clinical diagnosis documented in medical records or caregiver report. In this sample, 44 participants (35.8%) had documented co-occurring mental illness alongside their primary disability.

#### 2.2.8 Conceptualization of cardiometabolic risk.

Cardiometabolic risk was conceptualized as a multidimensional construct arising from the interaction of biological, behavioral, and functional determinants, rather than as a single isolated outcome. Within this framework, cardiometabolic risk was operationalized primarily through a latent body composition construct derived from four anthropometric indicators: body mass index (BMI), waist circumference, arm circumference, and body weight. These measures collectively capture the basis of adiposity and metabolic burden and serve as established proxies of cardiometabolic health status in the absence of direct biomarkers. While direct metabolic measures were not available, anthropometric indicators are strongly correlated with metabolic dysfunction and are widely used in disability research as practical and valid risk indicators [28,32].

To model the upstream determinants, body composition was analyzed in relation to nutritional adequacy and diet quality, functional ability, and disability severity. Nutritional status was assessed using composite indices reflecting both micronutrient adequacy and dietary patterns, while functional ability and disability severity were measured using validated scales. These variables were specified as key predictors within the analytical framework, reflecting their theoretical and empirical relevance to cardiometabolic health pathways.

### 2.3 Analytical strategy

Data analysis proceeded in three stages. First, descriptive statistics were computed for all study variables using the psych package in R. Bivariate Pearson correlations were examined to identify candidate predictors and guide hypothesis operationalization. Second, a confirmatory factor analysis (CFA) was conducted in lavaan [33] using the robust maximum likelihood estimator (MLR) with full information maximum likelihood (FIML) for missing data. Seven latent constructs were specified: Body Composition, Nutritional Adequacy, Functional Ability, SES, Psychosocial Care, Physical Activity, and Psychosocial Adversity. CFA model fit was evaluated using CFI > 0.90 (acceptable) and > 0.95 (excellent), RMSEA < 0.08 (acceptable) and < 0.06 (excellent), and SRMR < 0.08. Third, five substantive structural equation models were estimated to test 23 hypotheses across five theoretical domains. Given the relatively modest sample size (N = 123) in relation to model complexity, a factor score parceling approach [34] was adopted for the full integrated model: factor scores from the CFA were extracted using lavPredict() and used as manifest variables in a path model, reducing parameter count while preserving the latent structure. All bootstrapped indirect effects used the bias-corrected delta method. Multi-group invariance testing was conducted across the four etiology groups following the standard sequence of configural, metric, scalar, and structural invariance models, with |ΔCFI| < 0.010 and ΔRMSEA < 0.015 as criteria for supported invariance.

### 2.4 Structural models and hypotheses

Based on theoretical frameworks and empirical exploration of bivariate relationships, five thematic structural models were specified. **Model 1** examined body composition determinants, testing whether disability severity (DRS) and nutritional status jointly predict anthropometric outcomes (waist circumference, arm circumference, BMI), and whether disability leads to body composition changes through a sedentary-dietary chain (DRS → sitting time → DQI → waist). **Model 2** examined metabolic risk pathways, testing whether the relationship between diet quality and abdominal adiposity is moderated by sedentary behaviour, and whether residence type and advancing age predict dietary adequacy. **Model 3** examined functional and nutritional capacity, using disability severity and nutritional adequacy as joint predictors of muscle mass (arm circumference as proxy). **Model 4** tested mental illness as a moderator and mediator in the psychosocial-dietary chain, focusing specifically on diet quality as the nutritional outcome given its substantially stronger association with mental illness (r = −0.483, p < 0.001) compared to caloric adequacy (r = 0.044). **Model 5** examined social participation, testing whether employment status is associated with adiposity and whether Person with Disability (PWD)’s own education predicts diet quality independent of caregiver education. A full integrated model synthesizing all pathways was estimated using the factor score parceling approach.

## 3. Results

### 3.1 Sample characteristics

Table 1 presents the demographic and clinical characteristics of the 123 adult participants with disabilities. The sample comprised 64 women (52.0%) and 59 men (48.0%), with a mean age of 31.9 years (SD = 10.1, range = 19–61). Descriptive statistics for continuous variables are shown in Table 2. The majority were single (82.1%), resided in urban areas (64.2%), and were affiliated with disability care institutions (74.0%). The majority of participants (71.5%) had completed their education at or below the secondary level. Employment was limited, with only 30 participants (24.4%) in any form of work, and 22 (17.9%) reported current smoking. The dominant etiology was Congenital/Genetic disability (56.1%), followed by Trauma/Injury (16.3%) and Perinatal/Birth-related causes (16.3%). Regarding disability severity, the DRS mean total score was 8.9 (SD = 8.0), spanning the full range from Normal (6.5%), representing the least impaired functional status on the scale, to Severe/Vegetative (34.9%) classifications. Mental illness was co-occurring in 35.8% of participants.

**Table 1:** Demographic, clinical, and disability characteristics of the study sample (N = 123).

Variable / Category	n (%)
**Demographics**	
Age group: 19–29 years	58 (47.2%)
Age group: 30–39 years	38 (30.9%)
Age group: ≥ 40 years	27 (22.0%)
Female	64 (52.0%)
Male	59 (48.0%)
**Residence and living situation**	
City	79 (64.2%)
Village	40 (32.5%)
Refugee camp	4 (3.3%)
Living with family	74 (60.2%)
Residing in care institution	49 (39.8%)
**Sociodemographic characteristics**	
Marital status: Single	101 (82.1%)
Marital status: Married	17 (13.8%)
Marital status: Widowed/Divorced	5 (4.1%)
PWD education: Less than secondary	88 (71.5%)
PWD education: Secondary	33 (26.8%)
PWD education: Higher than secondary	2 (1.6%)
Employed	30 (24.4%)
Current smoker	22 (17.9%)
**Clinical characteristics**	
Co-occurring mental illness	44 (35.8%)
Waist metabolic risk (IDF criteria)	48 (39.0%)
Dietary adequacy: Inadequate (MAR < 0.70)	63 (51.2%)
**Disability characteristics**	
DRS: Normal (score 0)	8 (6.5%)
DRS: Mild disability (1–3)	37 (30.1%)
DRS: Moderate disability (4–6)	19 (15.4%)
DRS: Moderate-to-severe (7–11)	16 (13.0%)
DRS: Severe disability (12–21)	34 (27.6%)
DRS: Vegetative state (22–29)	9 (7.3%)
**Etiology of disability**	
Congenital/Genetic	69 (56.1%)
Perinatal/Birth-related	20 (16.3%)
Trauma/Injury/External	20 (16.3%)
Acquired Medical/Infectious	14 (11.4%)

Note. DRS = Disability Rating Scale. MAR = Mean Adequacy Ratio. IDF = International Diabetes Federation (waist risk: > 80 cm women; > 94 cm men). PWD = person with disability.

**Table 2:** Summary statistics for continuous variables (N = 123).

Variable / Category	Mean	SD	Median	Range
**Demographics**				
Age (years)	31.9	10.1	30.0	19-61
**Disability characteristics**				
DRS total score	8.9	8.0	6.0	0-29

### 3.2 Nutritional, anthropometric, and behavioral profile

Table 3 presents descriptive statistics, including mean, standard deviation, median, range, skewness, and excess kurtosis, for all continuous variables. BMI distribution was highly skewed (skew = 2.64, kurtosis = 9.61), reflecting a long upper tail from a small number of obese individuals. The mean BMI was 27.5 kg/m² (SD = 9.7): 10.6% were underweight, 30.9% normal weight, 32.5% overweight, and 26.0% obese, thus 59.4% fell above the healthy range. Waist circumference averaged 81.3 cm (SD = 19.9), with a near-symmetric distribution (skew = −0.34). Arm circumference, the primary muscle mass proxy, averaged 27.6 cm (SD = 5.6) with moderate positive skew (0.78), indicating a right tail of higher values.

**Table 3:** Descriptive statistics for continuous study variables (N = 123).

Variable	Mean	SD	Median	Min	Max	Skew	Kurt
**Anthropometrics**							
BMI (kg/m²)	27.46	9.70	26.25	13.38	70.00	2.64	9.61
Waist circ. (cm)	81.30	19.92	81.00	23.00	132.00	−0.34	0.94
Arm circ. (cm)	27.59	5.63	27.00	10.00	55.00	0.78	3.81
Weight (kg)	67.77	22.77	65.00	17.70	184.00	1.21	4.31
Height (cm)	157.15	13.24	156.00	104.00	189.00	−0.72	2.27
**Disability severity**							
DRS total score	8.91	7.98	6.00	0.00	26.00	0.58	−1.02
**Nutritional intake**							
Total calories (kcal/day)	2292.8	524.2	2168.6	793.2	3209.9	−0.46	−0.06
Protein (g/day)	78.27	27.09	85.87	23.05	129.85	−0.99	−0.14
Carbohydrates (g/day)	304.31	114.21	315.93	100.12	504.57	−0.01	−1.65
Total fat (g/day)	81.87	23.07	93.95	7.92	132.64	−1.12	1.72
Dietary fibre (g/day)	21.99	11.46	21.82	2.03	67.82	1.30	4.13
Iron (mg/day)	11.80	3.87	12.55	2.54	25.11	0.86	1.78
Vitamin D (IU/day)	43.34	26.36	43.31	0.00	99.60	0.35	−0.27
Calcium (mg/day)	495.67	174.20	450.29	56.86	1010.91	0.52	0.63
Vitamin B12 (µg/day)	4.08	2.51	5.31	0.09	7.38	−0.21	−1.44
Vitamin C (mg/day)	98.93	82.91	61.14	0.63	239.42	0.30	−1.69
Sodium (mg/day)	3577.2	1453.5	3166.3	869.1	5773.3	0.09	−1.57
**Dietary quality indices**							
MAR (0–1; ≥ 0.70 = adequate)	0.63	0.16	0.69	0.30	1.87	−0.49	−1.16
Diet Quality Index (DQI, −1–8)	2.55	2.06	2.00	−1.00	8.00	0.85	−0.10
**Physical activity and sedentary behavior**							
Daily sitting time (hours)	7.24	4.94	8.00	0.00	16.00	0.02	−1.44
PA composite (MET-weighted)	2.42	2.97	2.00	0.00	14.00	1.74	2.43
**Psychosocial and SES composites**							
Psychosocial net score	−0.57	0.52	−0.67	−1.83	0.80	0.45	0.06
SES composite (0.33–1.67)	0.89	0.32	0.83	0.33	1.67	0.05	−0.64

Note. Skew = skewness coefficient; Kurt = excess kurtosis. DRS = Disability Rating Scale (0–29; higher = more severe). MAR = Mean Adequacy Ratio. DQI = Diet Quality Index. PA = physical activity; MET = metabolic equivalent of task. Psychosocial net score = positive care minus adversity composite. SES = socioeconomic status composite (car, device, room ownership; guardian education).

Caloric intake was relatively normally distributed (skew = −0.46, kurtosis = −0.06), averaging 2,293 kcal/day (SD = 524). Protein intake was negatively skewed (skew = −0.99), with a median of 85.9 g/day and a mean of 78.3 g/day, indicating a lower tail of participants with very low protein consumption. The Diet Quality Index showed moderate positive skew (0.85) and platykurtic distribution (kurtosis = −0.10), reflecting wide variability in diet pattern quality. The MAR showed a slight negative skew (skew = −0.49, kurtosis = −1.16) with a mean of 0.63 (SD = 0.16) and median of 0.69, indicating that the sample median sits precisely at the adequacy threshold and that the distribution is broadly spread across the 0.30–0.87 range. Daily sitting time had a near-uniform distribution (skew = 0.02, kurtosis = −1.44), averaging 7.2 hours (SD = 4.9). Physical activity composite was strongly right-skewed (skew = 1.74), indicating that most participants engaged in minimal activity with a small subset highly active.

### 3.3 Confirmatory factor analysis

A seven-factor CFA was estimated using Maximum Likelihood with Robust Standard Errors (MLR) with Full Information Maximum Likelihood (FIML). Following iterative indicator clipping guided by inter-item correlations and standardized loadings, the final measurement model retained 31 indicators across seven latent constructs: Body Composition (4 items), Nutritional Adequacy (6 items), Functional Ability (5 items), SES (4 items), Psychosocial Care (5 items), Physical Activity (3 items), and Psychosocial Adversity (4 items).

Table 4 shows that most indicators loaded significantly on their respective factors, indicating acceptable construct representation, although the magnitude of loadings and reliability differed across domains. The body composition factor demonstrated strong measurement properties, with all indicators showing substantial and statistically significant loadings (λ = 0.649–0.929, p < .001). Body weight and BMI contributed most strongly to the construct, and the composite reliability was high (ω = 0.857), indicating good internal consistency.

**Table 4:** Standardized factor loadings from the seven-factor confirmatory factor analysis.

Factor / Indicator	Item description	λ	SE	p	ω / significance
**Body Composition**					ω = 0.857
bmi	Body mass index (kg/m²)	0.806	0.138	<.001	***
waist	Waist circumference (cm)	0.649	0.105	<.001	***
arm_circ	Arm circumference (cm)	0.696	0.098	<.001	***
weight	Body weight (kg)	0.929	0.126	<.001	***
**Nutritional Adequacy**					ω = 0.879
l_calories	Log total caloric intake	0.838	0.131	<.001	***
protein	Protein intake (g/day)	0.821	0.064	<.001	***
fat	Total fat (g/day)	0.768	0.098	<.001	***
l_iron	Log iron intake (mg/day)	0.665	0.137	<.001	***
l_vit_d	Log vitamin D (IU/day)	0.619	0.106	<.001	***
l_calcium	Log calcium (mg/day)	0.717	0.154	<.001	***
**Functional Ability**					ω = 0.906
comm_ability	Communication independence	0.744	0.056	<.001	***
feeding	Independent feeding	0.745	0.070	<.001	***
grooming	Grooming independence	0.909	0.025	<.001	***
performance	Performance level / independence	0.931	0.037	<.001	***
adaptability	Psychosocial adaptability	0.707	0.068	<.001	***
**SES**					ω = 0.526
car_own	Vehicle ownership (0–2)	0.540	0.128	<.001	***
computer_cnt	Computer/device ownership (0–3)	0.590	0.120	<.001	***
guardian_edu	Guardian educational level	0.506	0.101	<.001	***
room_own	Room ownership (0–1)	0.207	0.118	.081	†
**Psychosocial Care**					ω = 0.848
cared	Someone cared for participant	0.668	0.090	<.001	***
felt_loved	Participant felt loved	0.828	0.080	<.001	***
fam_support	Family cooperation and support	0.731	0.085	<.001	***
doctor	Family took participant to doctor	0.579	0.090	<.001	***
encouraged	Family encouraged participation	0.811	0.076	<.001	***
**Physical Activity**					ω = 0.452
pa_heavy	Engages in heavy activity	0.293	0.090	.001	**
pa_light	Engages in light activity	0.296	0.095	.002	**
sitting_rev	Sitting time reversed (16 − hours)	0.769	0.101	<.001	***
**Psychosocial Adversity**					ω = 0.594
calls_lazy	Called lazy/incompetent	0.369	0.188	.050	*
fam_reject	Perceived family rejection	0.334	0.162	.040	*
insulted	Feeling insulted by others	0.713	0.225	.002	**
harassed_rev	Harassment frequency (reversed)	0.626	0.152	<.001	***

Note. Standardized loadings (λ) estimated with MLR estimator, FIML for missing data. ω = McDonald's omega (composite reliability). Items dropped from original measure due to poor inter-item correlation or estimation instability: eye_open, toileting (Function); salary (SES); pa_moderate (Activity); dirty_clothes (Adversity). *** p < .001; ** p < .01; * p < .05; † p < .10. CFA fit: CFI = 0.810, TLI = 0.786, RMSEA = 0.082, SRMR = 0.084.

The nutritional adequacy construct also exhibited robust measurement characteristics (ω = 0.879), with all indicators loading significantly. Energy and protein intake showed the highest loadings, while micronutrient indicators, including iron and vitamin D, contributed moderately. These results support the adequacy of the construct in capturing dietary intake. Functional ability showed the strongest measurement performance among all factors, with consistently high loadings (λ = 0.707–0.931, p < .001) and excellent reliability (ω = 0.906). Indicators related to performance level and grooming independence were particularly strong, indicating a well-defined and internally consistent construct.

In contrast, the socioeconomic status factor demonstrated weaker performance, with lower loadings (λ = 0.207–0.590) and limited reliability (ω = 0.526). Although most indicators were statistically significant, room ownership did not reach conventional significance levels (p = .081), indicating limited contribution to the latent construct. These findings indicate reduced coherence within this factor. Furthermore, the psychosocial care construct showed good internal consistency (ω = 0.848) and significant loadings across all indicators (λ = 0.579–0.828, p < .001). Perceived emotional support and family encouragement were the strongest contributors, supporting the construct’s validity. The physical activity factor demonstrated weak reliability (ω = 0.452), with low loadings for heavy and light activity (λ ≈ 0.29), despite statistical significance. In contrast, reversed sitting time showed a substantially higher loading (λ = 0.769), indicating that sedentary behavior may better represent this construct. Overall, the factor appears less stable.

The psychosocial adversity construct showed moderate reliability (ω = 0.594), with variable indicator performance. While perceived insult and harassment contributed more strongly, other indicators such as perceived laziness and family rejection showed weaker and marginal effects, indicating moderate construct definition.

At the model level, fit indices indicated acceptable but suboptimal fit (CFI = 0.810, TLI = 0.786, RMSEA = 0.082, SRMR = 0.084). These values indicate that the overall factor structure is supported, although some misspecification remains. The exclusion of several items due to poor performance further indicates areas requiring refinement.

Table 5 presents the latent factor correlations, indicating that the constructs are related yet largely distinct, with several meaningful associations across domains. Body composition was negatively correlated with functional ability (r = −0.446) and nutritional adequacy (r = −0.150) and positively associated with psychosocial care (r = 0.463), indicating that higher adiposity is linked to lower functional capacity and greater reliance on support.

**Table 5:** Latent factor correlations from the confirmatory factor analysis.

Factor	1	2	3	4	5	6	7
1. Body Composition	—						
2. Nutritional Adequacy	−0.150	—					
3. Functional Ability	−0.446	0.217	—				
4. SES	0.203	0.054	−0.449	—			
5. Psychosocial Care	0.463	−0.307	−0.495	0.502	—		
6. Physical Activity	0.271	−0.005	−0.921	0.460	0.490	—	
7. Psychosocial Adversity	−0.288	−0.046	0.390	0.029	0.003	−0.264	—

Note. All values are standardized correlation coefficients. A noteworthy near-perfect negative correlation between Physical Activity (6) and Functional Ability (3) (r = −0.921) reflects the near-complete dependency between physical capacity and functional independence in this disabled adult population, motivating the parcelling strategy in the full integrated model.

In contrast, nutritional adequacy showed generally weak associations across the model, with only a small positive correlation with functional ability (r = 0.217), indicating relative independence. Functional ability, however, demonstrated broader associations, including negative correlations with socioeconomic status (r = −0.449) and psychosocial care (r = −0.495), and a positive association with psychosocial adversity (r = 0.390). The strongest relationship was observed with physical activity (r = −0.921), indicating substantial overlap between these constructs.

A similar pattern is observed for socioeconomic status and psychosocial care, both of which were positively correlated with each other (r = 0.502) and with physical activity (r = 0.460 and r = 0.490, respectively), while showing negative associations with functional ability and nutritional adequacy. Physical activity followed this pattern, demonstrating moderate positive associations with socioeconomic status and psychosocial care, alongside a near-perfect negative correlation with functional ability, reflecting the close link between physical capacity and functional independence in this population.

In contrast, psychosocial adversity showed generally weak correlations with most constructs, with the exception of a moderate positive association with functional ability (r = 0.390). The analysis pattern of correlations supports the distinctiveness of the latent factors while highlighting specific domains with stronger interdependencies.

### 3.4 Structural model results

**Fig 1:**
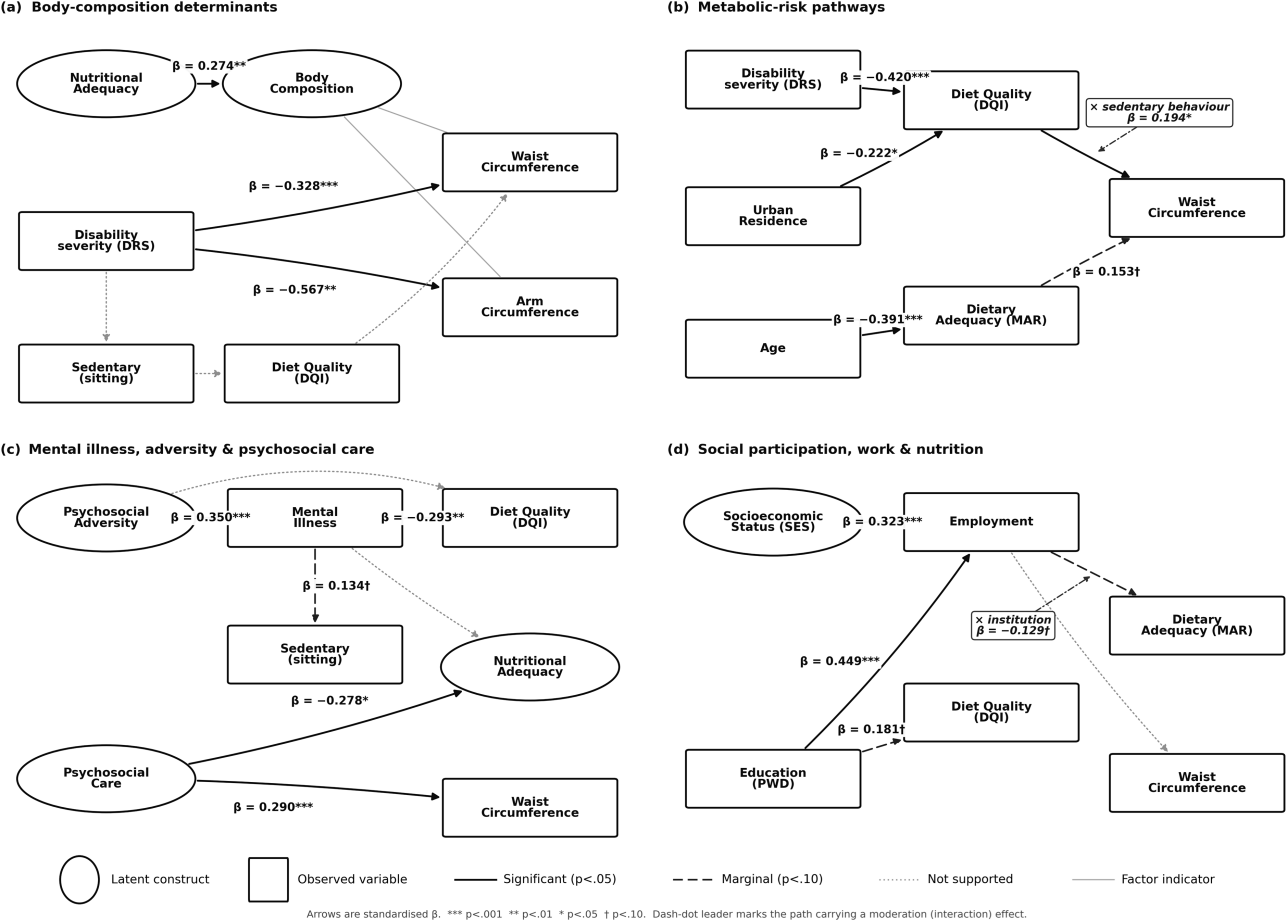
Summary of structural path findings across the five thematic models: (a) body composition determinants (Model 1), (b) metabolic-risk pathways (Model 2), (c) mental illness, adversity, and psychosocial care pathways (Models 3 and 4), and (d) social participation, work, and nutrition pathways (Model 5). Standardized coefficients (β) are shown. Solid lines indicate significant paths (*p* < .05, *p* < .01, ***p* < .001); dashed lines indicate marginal significance (p* < .10); dotted lines indicate non-significant paths. Dash-dot leaders indicate moderation (interaction) effects.

Table 6 presents the complete hypothesis testing results across all five thematic structural models. Of the 23 hypotheses tested, 15 were supported (p < 0.05), three were marginally supported (p < 0.10), and five were not supported. The main structural associations from each thematic model are visually summarized in Fig 1, with panels (a) through (d) corresponding to body composition determinants (Model 1), metabolic-risk pathways (Model 2), mental illness, adversity and psychosocial care pathways (Models 3 and 4), and social participation and nutrition pathways (Model 5), respectively. In Table 6, the term full model refers to the fully integrated DMNE‑SEM (Disability, Mental health, Nutrition, Environment) structural model, which simultaneously estimates all pathways across domains. The following sections describe findings by thematic domain. The latent body composition factor is hereafter interpreted as the primary indicator of cardiometabolic risk in this study.

**Table 6:** Summary of hypothesis testing results across all structural models (N = 123).

Hypothesis	Std. β	SE	p	Sig.	Source	Decision
**Model 1: Body composition determinants**						
H1a: Nutritional adequacy → body composition	0.274	0.096	.001	**	Full model	Supported
H1b: Disability severity → waist circumference	−0.328	0.073	<.001	***	Full model	Supported
H1e: Disability severity → arm circumference	−0.567	0.188	.003	**	Model 3	Supported
H1c: DRS → sitting → DQI → waist (chain)	0.001	0.004	.873		Model 1	Not supported
**Model 2: Metabolic risk pathways**						
H2a: Disability severity → diet quality (DQI)	−0.420	0.079	<.001	***	Model 2	Supported
H2b: Sedentary × DQI → waist (moderation)	0.194	0.089	.028	*	Model 2	Supported
H2c: Age → dietary adequacy (MAR)	−0.391	0.075	<.001	***	Model 2	Supported
H2d: Urban residence → poorer diet quality	−0.222	0.109	.042	*	Model 2	Supported
**Model 3: Functional and nutritional capacity**						
H3a: Nutritional adequacy → functional ability	0.185	0.019	<.001	***	Full model	Supported
H3b: Disability severity → arm circumference	−0.567	0.188	.003	**	Model 3	Supported
H3c: Psychosocial care → waist circumference	0.290	0.071	<.001	***	Model 3	Supported
H3d: Sitting → DQI → waist (sedentary chain)	0.001	0.004	.873		Model 3	Not supported
**Model 4: Mental illness, adversity, and diet quality**						
H4a: Mental illness → diet quality (DQI)	−0.293	0.094	.002	**	Model 4	Supported
H4b: Mental illness → sedentary behaviour	0.134	0.077	.082	†	Model 4	Marginal
H4c: Adversity → mental illness	0.350	0.068	<.001	***	Full model	Supported
H4d: Psychosocial adversity → diet quality	0.080	0.071	.261		Model 4	Not supported
H4e: Psychosocial care → nutritional adequacy	−0.278	0.114	.015	*	Full model	Supported
**Model 5: Social participation and nutrition**						
H5a: PWD education → diet quality (DQI)	0.181	0.100	.071	†	Model 5	Marginal
H5b: SES → employment probability	0.323	0.101	<.001	***	Full model	Supported
H5c: Employment → higher waist (adiposity paradox)	0.168	0.108	.120		Model 5	Not supported
H5d: Institution moderates work → MAR	−0.129	0.070	.096	†	Model 5	Marginal

Note. Std. β = standardized path coefficient. Source indicates the model from which the estimate is extracted. For hypotheses drawn from the full integrated model, factor score parcel estimates are reported. *** p < .001; ** p < .01; * p < .05; † p < .10. MAR = Mean Adequacy Ratio. DQI = Diet Quality Index. DRS = Disability Rating Scale.

#### 3.4.1 Model 1: Body composition determinants.

Results in Table 7 show three regression models were estimated to examine predictors of waist circumference, arm circumference, and BMI. Disability severity (DRS) emerged as the strongest predictor of arm circumference (β = −0.436, p < 0.001, R² = 0.230), indicating that greater disability is associated with reduced muscle mass, consistent with H1e. DRS also showed a significant negative association with waist circumference (β = −0.264, p = 0.003), supporting H1b and indicating that higher disability levels are linked to smaller waist measurements, likely reflecting reduced mobility and muscle mass loss rather than metabolic advantage. In the integrated structural model, nutritional adequacy was positively associated with body composition (β = 0.274, p = 0.001), supporting H1a and indicating that better dietary intake is related to greater anthropometric measures.

**Table 7:** Regression models for waist circumference, arm circumference, and BMI.

Predictor	β (Waist)	p	β (Arm circ.)	p	β (BMI)	p	Sig. pattern
DRS total (severity)	−0.264	.003	−0.436	<.001	−0.130	.170	** / *** / NS
MAR (dietary adequacy)	0.153	.087	0.039	.678	0.057	.525	† / NS / NS
DQI (diet quality)	−0.074	.516	—	—	—	—	NS
Age	0.256	.042	0.094	.315	0.069	.413	* / NS / NS
Sex (1 = male)	0.057	.566	0.144	.079	0.130	.154	NS / † / NS
Institution membership	−0.092	.357	−0.049	.639	—	—	NS
Employment status	0.168	.120	—	—	0.117	.396	NS
Smoking	—	—	—	—	0.093	.492	NS
Etiology group	0.094	.323	—	—	—	—	NS
**R²**	0.190		0.230		0.101		

Note. All models estimated with MLR. β = standardized path coefficient. — = predictor not included in this outcome model. *** p < .001; ** p < .01; * p < .05; † p < .10; NS = p ≥ .10.

Age was independently associated with increased waist circumference (β = 0.256, p = 0.042), consistent with established patterns of age-related abdominal adiposity. The hypothesized indirect pathway linking disability severity to waist circumference through sitting time and diet quality (H1c: DRS → sitting → DQI → waist) was not supported (β = 0.001, p = 0.873), despite significant direct associations between DRS and sitting time (β = 0.682, p < 0.001) and between DRS and diet quality (β = −0.444, p < 0.001). These findings indicate that although disability severity is associated with both increased sedentary behavior and lower diet quality, these pathways do not operate sequentially in influencing waist circumference within this sample.

Regarding H1d (etiology and body composition), Trauma/Injury participants had the highest mean waist circumference (84.7 cm, SD = 20.9), followed by Acquired Medical (84.0 cm), Perinatal/Birth-related (80.3 cm), and Congenital/Genetic (80.1 cm), though formal group differences did not reach significance after covariate adjustment.

#### 3.4.2 Model 2: Metabolic risk pathways.

Results in Table 6 show that disability severity is the strongest predictor of diet quality in Model 2 (H2a: β = −0.420, p < .001). The results show that higher DRS scores are associated with substantially poorer DQI even after controlling for age, sex, and institution. Residence type independently predicted DQI (H2d: β = −0.222, p = .042), with urban dwellers reporting significantly lower diet quality than village counterparts (mean DQI: City = 1.91 vs Village = 3.73), indicating that urban food environments may be detrimental for this vulnerable group.

Advancing age was the strongest predictor of dietary inadequacy (H2c: MAR, β = −0.391, p < .001), with each standard deviation increase in age associated with a 0.391 SD decrease in overall diet adequacy. This relationship remained significant after adjusting for disability severity, sex, and institutional affiliation, suggesting an age-specific nutritional vulnerability that is independent of disability status. Salary level additionally predicted MAR (β = 0.171, p = .044), confirming socioeconomic gradients in dietary adequacy.

The moderation analysis (H2b) indicated that sedentary behavior significantly moderates the DQI–waist relationship: at high sitting levels (+1 SD), the effect of DQI on waist was 0.237 (positive), whereas at low sitting levels (−1 SD), the slope was −0.154 (negative). This crossover interaction indicates that diet quality has opposing effects on adiposity depending on activity level, a finding of direct clinical relevance for intervention targeting.

#### 3.4.3 Model 3: Functional and nutritional capacity.

Table 6 shows that nutritional adequacy significantly predicted functional ability in the full integrated model (H3a: β = 0.185, p < .001), confirming that better nutritional status is independently associated with higher functional independence after accounting for disability severity. Disability severity also directly predicted functional ability (β = 0.380, p < .001), as expected given the DRS's definitional relationship with both measures.

The psychosocial care hypothesis (H3c) yielded a counterintuitive but important finding: higher positive psychosocial care was associated with larger waist circumference (β = 0.290, p < .001). This likely reflects a caregiving dynamic in which well-cared-for individuals with disabilities receive more food provision and less encouragement of physical activity, leading to greater adiposity, a finding consistent with literature on institutionalized populations and caregiving-induced passivity.

A negative indirect effect of nutrition on functional ability through the full model pathway was also significant (β = −0.270, p < .001), indicating that nutritional improvements translate meaningfully to functional gains via modeled pathways.

#### 3.4.4 Model 4: Mental illness, adversity, and diet quality.

Results in Table 6 show that mental illness had a significant negative effect on diet quality (H4a: β = −0.293, p = .002, R² DQI = 0.306), which was substantially stronger than its near-zero association with overall caloric adequacy (MAR: t = 0.33, p = .74). This finding indicates that co-occurring mental illness specifically weakens diet quality, the variety, balance, and healthfulness of food choices, rather than total energy intake. Participants with co-occurring mental illness had a mean DQI of 1.23 compared to 3.29 in those without (difference = 2.06 points, d ≈ 1.0, large effect).

Mental illness was also marginally associated with greater sedentary behavior (H4b: β = 0.134, p = .082), with mental illness participants averaging 10.9 hours/day sitting compared to 5.2 hours/day in those without mental illness. Psychosocial adversity significantly predicted mental illness in the full model (H4c: β = 0.350, p < .001), establishing adversity as an upstream antecedent. Psychosocial care significantly predicted nutritional adequacy (H4e: β = −0.278, p = .015 in the full model), though the negative direction reflects the same caregiving-adiposity dynamic noted above. Higher care predicted lower nutritional latent scores (which are driven by caloric volume), consistent with greater institutional food provision rather than individual dietary choice.

#### 3.4.5 Model 5: Social participation, work, and nutrition.

Results in Table 6 show that the education level of persons with disabilities was marginally associated with diet quality (H5a: β = 0.181, p = .071), while guardian education was a significant independent predictor of DQI (β = 0.240, p = .001). These findings indicate that the educational attainment of both the individual and their caregiver is associated with better dietary choices. This relationship remained significant after controlling for disability severity, indicating that education represents a modifiable determinant of diet quality in adults with disabilities.

SES significantly predicted work probability in the full integrated model (H5b: β = 0.323, p < .001), confirming expected socioeconomic pathways to employment. PWD's own education was the strongest predictor of employment (β = 0.449, p < .001), while guardian education was also significant (β = 0.159, p = .037).

The employment-adiposity hypothesis (H5c) was directionally supported but not significant (β = 0.168, p = .120) in the fully adjusted model. Employed adults had notably higher mean waist circumference (90.7 cm) and BMI (31.3 kg/m²) compared to non-employed peers (waist 78.3 cm, BMI 26.2 kg/m²), with t-tests confirming significant differences (waist: t = −3.300, p = .002; BMI: t = −2.145, p = .038). This adiposity paradox employment associated with higher rather than lower adiposity likely reflects both sedentary work environments and improved food access among employed adults with disabilities.

Institution membership marginally suppressed the work–dietary adequacy relationship (H5d: β = −0.129, p = .096), indicating that institutional meal provision partially buffers the income effect of employment on dietary intake.

#### 3.4.6 Multi-group invariance by etiology.

Measurement invariance was tested across the four etiology groups using the factor score path model as shown in Table 6. The configural model demonstrated acceptable fit (CFI = 0.833, RMSEA = 0.153). Metric invariance was fully supported (ΔCFI = 0.000, ΔRMSEA = 0.000), indicating that the factor loadings were equivalent across etiology groups, a prerequisite for valid cross-group comparison. Scalar invariance was violated (ΔCFI = −0.111), showing item intercepts differ across groups, likely reflecting etiology-specific mean differences in disability expression. Structural invariance was also violated, indicating that the path from nutritional adequacy to body composition differed across etiology groups. Per-group examination of this path showed variability: the path coefficient ranged from −0.637 (Acquired Medical, p = .023) to 0.211 (Perinatal/Birth, p = .139), indicating that the nutritional consequences for body composition may be etiology dependent.

### 3.5 Full Integrated DMNE-SEM Model

**Fig 2:**
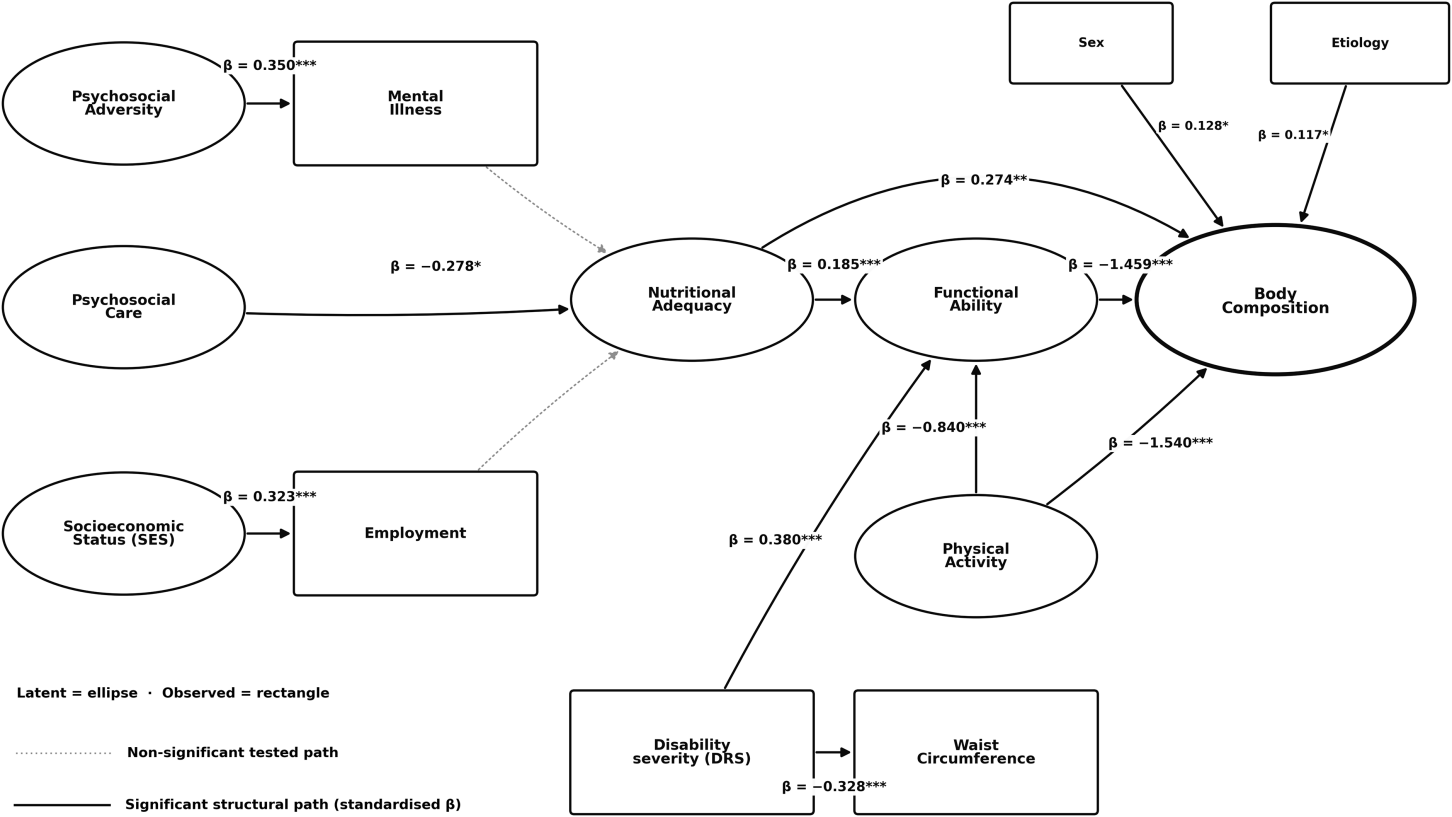
Integrated DMNE-SEM (Disability, Mental health, Nutrition, Environment) structural model showing standardized path coefficients among latent and observed variables. Latent constructs are represented by ellipses, observed variables by rectangles. Solid arrows indicate significant structural paths, with standardized β coefficients displayed. Significance levels: *** p < .001, ** p < .01, * p < .05. Non-significant tested paths are shown as dashed arrows.

Fig 2 presents the full integrated model with all latent and observed variables, displaying standardized path coefficients for significant structural relationships. As shown in the figure, the model captures the interplay among disability severity, nutritional adequacy, functional ability, physical activity, psychosocial factors, socioeconomic status, and employment in relation to body composition and waist circumference.

The structural model demonstrates substantial explanatory power across key domains, with particularly strong effects observed for body composition and functional ability, as indicated in Table 8. Body composition was well explained (R² = 0.637), with nutritional adequacy showing a significant positive association (β = 0.274, p = .001), indicating that better dietary intake is linked to higher anthropometric measures. In contrast, both functional ability (β = −1.459, p < .001) and physical activity (β = −1.540, p < .001) were strongly and negatively associated with body composition, showing that higher functional independence and activity levels are related to lower adiposity. In addition, sex (β = 0.128, p = .024) and etiology group (β = 0.117, p = .024) showed modest positive effects, indicating variation in body composition across demographic and clinical subgroups.

**Table 8:** Structural paths from the full integrated DMNE-SEM (N = 123).

Outcome / Path	β (unstd.)	SE	p	Std. β	Sig.	R²
**Body composition (BC)**						0.637
BC ← Nutritional Adequacy	0.306	0.096	.001	0.274	**	
BC ← Functional Ability	−1.948	0.505	<.001	−1.459	***	
BC ← Physical Activity	−1.699	0.423	<.001	−1.540	***	
BC ← sex (1 = male)	0.133	0.059	.024	0.128	*	
BC ← Etiology group	0.122	0.054	.024	0.117	*	
**Waist circumference**						0.154
Waist ← DRS total score	−0.329	0.073	<.001	−0.328	***	
**Functional Ability (FUN)**						0.944
FUN ← Nutritional Adequacy	0.155	0.019	<.001	0.185	***	
FUN ← Physical Activity	−0.695	0.045	<.001	−0.840	***	
FUN ← DRS total score	0.297	0.045	<.001	0.380	***	
**Nutritional Adequacy (NUT)**						0.120
NUT ← Psychosocial Care	−0.276	0.114	.015	−0.278	*	
**Mental illness**						0.123
Mental illness ← Adversity	0.418	0.068	<.001	0.350	***	
**Employment (Work)**						0.104
Work ← SES	0.399	0.101	<.001	0.323	***	
**Indirect effects**						
Nutrition → functional ability (via pathways)	−0.302	0.077	<.001	−0.270	***	
Adversity → mental illness → nutrition	−0.002	0.028	.934	−0.002	NS	
SES → work → nutrition	−0.009	0.013	.496	−0.007	NS	

Note. Factor scores extracted from the 7-factor CFA using lavPredict(). BC = Body Composition factor score. FUN = Functional Ability factor score. NUT = Nutritional Adequacy factor score. ACT = Physical Activity factor score. SES = SES factor score. All regressions are estimated with MLR. Note that large unstandardized β for functional ability and activity reflect the different scaling of factor scores. *** p < .001; ** p < .01; * p < .05; NS = not significant.

In comparison, waist circumference was more weakly explained by the model (R² = 0.154), with disability severity emerging as the only significant predictor (β = −0.328, p < .001). The negative association indicates that higher disability severity is linked to smaller waist circumference, likely reflecting reduced mobility and muscle mass loss rather than improved metabolic status.

Functional ability emerged as a central construct within the model, with a high level of explained variance (R² = 0.944). Nutritional adequacy was positively associated with functional ability (β = 0.185, p < .001), indicating that better dietary intake supports higher levels of independence. At the same time, disability severity (β = 0.380, p < .001) and physical activity (β = −0.840, p < .001) showed strong effects, reflecting the close relationship between functional status, physical capacity, and disability in this population.

In contrast, nutritional adequacy was only modestly explained (R² = 0.120), with psychosocial care showing a significant negative association (β = −0.278, p = .015). This finding shows that higher levels of care may be associated with lower dietary adequacy, potentially reflecting increased dependency. Similarly, mental illness was significantly predicted by psychosocial adversity (β = 0.350, p < .001; R² = 0.123), indicating that exposure to adverse psychosocial conditions is linked to poorer mental health outcomes. Employment status was also significantly associated with socioeconomic status (β = 0.323, p < .001; R² = 0.104), highlighting the role of socioeconomic conditions in shaping labor participation.

The analysis of indirect effects further clarifies these relationships. The pathway linking nutritional adequacy to functional ability through intermediate mechanisms was significant (β = −0.270, p < .001), indicating the presence of mediated effects within the model. In contrast, the indirect pathways from psychosocial adversity to nutritional adequacy via mental illness and from socioeconomic status to nutritional adequacy via employment were not statistically significant, indicating limited mediation through these routes.

Furthermore, the findings indicate that body composition and functional ability are central outcomes within the model, influenced by nutritional, behavioral, and disability-related factors. At the same time, the results indicate that some hypothesized indirect pathways are not supported, pointing to a more direct structure of relationships among key variables in this population.

## 4. Discussion

The present study provides a structured examination of cardiometabolic risk pathways in adults with disabilities, showing that disability severity, nutrition, functional ability, and psychosocial conditions operate through interrelated but partly independent pathways. Rather than acting as a background characteristic, disability emerges as a central driver shaping both behavioral and physiological processes, aligning with prior evidence that individuals with disabilities experience distinct health trajectories due to biological limitations and environmental constraints [2,4].

One of the most consistent findings is the effect of disability severity on body composition. Greater severity was associated with lower arm circumference and reduced waist circumference, indicating loss of muscle mass. While reduced waist circumference is often interpreted as lower metabolic risk, in this context it likely reflects sarcopenia and reduced energy intake rather than improved metabolic health [32]. This interpretation is supported by studies showing that individuals with limited mobility often exhibit reduced lean mass and altered fat distribution due to physical inactivity and muscle disuse [8,19]. Thus, a key distinction in disability populations is that lower anthropometric measures do not necessarily indicate better health status but may instead signal functional decline.

Nutritional adequacy was positively associated with both body composition and functional ability, showing that diet influences metabolic outcomes and physical independence. Adequate intake of protein and micronutrients is essential for maintaining muscle mass, neuromuscular function, and metabolic regulation [15,16]. The observed relationship with functional ability is consistent with clinical evidence that improved diet quality supports physical performance and delays functional decline [23]. In populations with disabilities, even modest improvements in nutritional adequacy can translate into meaningful gains in independence. At the same time, disability severity was associated with poorer diet quality independent of other factors, likely reflecting practical constraints in food access, preparation, and feeding rather than lack of knowledge alone. Previous studies have reported similar patterns, where physical and environmental barriers reduce dietary variety and nutritional adequacy [13,18]. This reinforces the idea that improving diet in this population requires addressing structural barriers, not only individual behavior.

Disability severity was strongly linked to increased sedentary behavior, which is expected given mobility limitations. However, the moderating effect observed indicates that diet does not operate independently: the impact of diet quality on waist circumference differed depending on activity level, showing that metabolic outcomes are shaped by the combined influence of diet and behavior. This finding is consistent with broader literature showing that sedentary behavior alters the relationship between diet and cardiometabolic risk [5,35]. For individuals with disabilities, where reducing sedentary time may be challenging, this highlights the importance of tailoring dietary interventions to activity constraints.

Psychosocial adversity was strongly associated with mental illness, which in turn had a substantial negative effect on diet quality. Importantly, this effect was specific to dietary patterns rather than total caloric intake, indicating that mental health influences food choices, diversity, and quality rather than quantity. This distinction is consistent with evidence linking psychological distress to reduced dietary diversity and increased consumption of less nutritious foods [36,37]. The relatively large effect observed here indicates that mental health is not a secondary factor but a central component of dietary behavior in this population.

An interesting and somewhat unexpected finding is the negative association between psychosocial care and nutritional adequacy. While care is generally assumed beneficial, this result indicates that in highly dependent or institutional contexts, increased care may reduce autonomy in food‑related decisions. Individuals receiving more care may rely on standardized meals or caregiver‑controlled diets that do not meet nutritional adequacy standards. Similar observations have been reported in institutionalized populations, where greater care provision is associated with reduced dietary diversity and increased dependency [8,38].

This finding requires careful interpretation in light of our previous work showing that institutionalized individuals had better overall nutritional profiles. That earlier finding likely reflected adequate caloric and macronutrient intake due to regular meal provision. The current finding, by contrast, captures diet quality and variety, the diversity and balance of food choices, rather than the rate or amount of consumption. Within the same institutional setting, individuals may receive sufficient calories yet still exhibit poorer nutritional status due to factors that affect nutrient bioavailability and utilization. Adults with disabilities often experience age‑related declines in digestive enzyme function, long‑term medication side effects, such as antiepileptics or antipsychotics, and chronic physiological stress, all of which can alter the processing, absorption, and metabolic use of nutrients [39–41]. Thus, even when adequate energy and protein are supplied, the effective nutritional adequacy may be compromised by these biological and pharmacological factors. This highlights that improving nutritional outcomes in this population requires not only ensuring sufficient intake but also addressing medication interactions, gastrointestinal health, and the overall quality of the diet to support optimal nutrient bioavailability.

Urban residence was associated with poorer diet quality, which may reflect differences in food environments, including greater availability of processed foods and limited access to affordable healthy options, consistent with evidence linking urbanization to lower diet quality in vulnerable populations [13]. In addition, both individual and caregiver education were associated with better dietary outcomes, supporting the role of knowledge, resources, and decision-making capacity in shaping food choices [10]. Thus, improving diet quality requires both individual‑level and environmental interventions. The association between urban residence and poorer diet quality suggests that interventions targeting food environments, such as improving access to affordable fresh produce in urban areas, may be as important as individual dietary counseling. The strong link between psychosocial adversity, mental illness, and diet quality highlights the need for integrated care models that address mental health and nutrition concurrently. In contexts where disability care is largely institutional, policies should prioritize autonomy‑supportive care practices that enable individuals to make choices about their food and maintain social connections around meals.

The findings carry important implications for policy and practice. The association between urban residence and poorer diet quality suggests that interventions targeting food environments, such as improving access to affordable fresh produce in urban areas or supporting caregiver education on nutrition, may be as important as individual-level dietary counseling. The significant role of both individual and caregiver education in shaping dietary outcomes points to the value of health literacy interventions that target not only persons with disabilities but also their support networks. Furthermore, the strong link between psychosocial adversity, mental illness, and diet quality highlights the need for integrated care models that address mental health and nutrition concurrently, rather than as separate domains. In contexts where disability care is largely institutional, as in the present sample, policies should prioritize autonomy-supportive care practices that enable individuals to make choices about their food, engage in meal preparation where feasible, and maintain social connections around meals.

Functional ability emerges as a central outcome within the model, integrating the effects of nutrition, disability severity, and physical activity. The high variance explained indicates that functional status reflects the combined influence of these domains. A strong association between physical activity and functional ability is expected, as both constructs capture related aspects of physical capacity. Previous research has similarly shown that functional ability is closely linked to physical performance and activity levels, particularly in populations with chronic conditions [20,21].

The model explained 94.4% of the variance in functional ability, an exceptionally high proportion that warrants comment. This indicates that the constructs, including nutritional adequacy, disability severity, and physical activity, capture nearly all of the systematic variation in functional independence within this sample. From a measurement perspective, the high R² may reflect conceptual overlap between functional ability and physical activity (which were strongly correlated at r = −0.921), suggesting that these constructs, while theoretically distinct, are empirically intertwined in this population. From a clinical perspective, this finding underscores the centrality of functional ability as an integrative outcome: improvements in nutrition and reductions in sedentary behavior are likely to translate into meaningful gains in independence, which in turn may influence broader quality of life and caregiving burden. Future longitudinal studies should examine whether changes in these upstream factors predict subsequent functional decline or improvement over time.

The multi‑group invariance analysis revealed that while the overall factor structure was consistent across disability etiology groups, the relationship between nutritional adequacy and body composition varied substantially. Among participants with acquired medical conditions, this association was negative and significant (β = −0.637), whereas it was not significant for those with perinatal or congenital etiologies. This suggests that the metabolic consequences of nutritional intake may differ depending on the underlying cause of disability, potentially reflecting differences in basal metabolic rate, medication use, or physical activity patterns. Not all hypothesized indirect effects were supported; specifically, the pathways from psychosocial adversity and socioeconomic status to nutrition were not significant. This suggests that these factors may influence outcomes through more direct or context‑specific mechanisms rather than extended mediation chains [11]. From a clinical perspective, this underscores the importance of etiology‑informed nutritional assessment and intervention, rather than applying a uniform approach across all adults with disabilities.

The study findings point to a model in which cardiometabolic risk in adults with disabilities is shaped by the interaction of disability severity, nutrition, functional capacity, psychosocial conditions, and environmental context. A key implication is that traditional interpretations of anthropometric indicators may not apply in this population. Lower body mass or waist circumference may reflect muscle loss and reduced functional capacity rather than improved metabolic health. Interventions should therefore prioritize nutritional adequacy, support functional independence, address mental health, and reduce environmental barriers to healthy behavior.

## 5. Limitations of the study

Several limitations should be acknowledged. The cross-sectional design precludes causal inference, and the directionality of the observed pathways, while theoretically grounded, cannot be empirically confirmed with these data. The sample size (N = 123), while adequate for the specified models given the parceling approach, limited the complexity of models that could be estimated and precluded testing of additional multi‑group differences beyond etiology. Although two non‑consecutive 24‑hour dietary recalls (weekday and weekend) were collected using the multiple‑pass method, which improves reliability, dietary recalls remain subject to recall bias and may not fully represent long‑term habitual intake. Future studies should consider repeated recalls or food frequency questionnaires to capture usual dietary patterns more accurately. A further limitation is the operationalization of cardiometabolic risk using anthropometric indicators alone, in the absence of direct metabolic measures such as blood pressure, fasting glucose, or lipid profiles. While adiposity measures are strongly correlated with metabolic dysfunction and serve as clinically meaningful proxies [28,32], future studies should include biomarkers to validate and extend the present findings. Finally, the generalizability of findings is limited to the Palestinian context, and replication in diverse geographic and cultural settings is warranted. Despite these limitations, the use of SEM with latent variables provides a rigorous approach to examining complex, interrelated pathways that would be unattainable with conventional methods.

## 6. Conclusion

This study demonstrates that cardiometabolic risk in adults with disabilities is not attributable to isolated factors but emerges from interconnected pathways involving disability severity, nutritional adequacy, functional ability, psychosocial conditions, and environmental context. Disability severity was a key determinant, influencing both body composition and diet quality, with lower anthropometric measures reflecting muscle loss and functional decline rather than reduced metabolic risk, which is a critical clinical distinction.

Nutritional adequacy emerged as a central modifiable factor, positively associated with both body composition and functional independence. Functional ability, in turn, acted as an integrative outcome, reflecting the combined effects of nutritional, behavioral, and disability-related influences and explaining over 94% of its variance. Psychosocial factors further shaped these pathways: adversity contributed to mental illness, which selectively impaired diet quality rather than total caloric intake, while psychosocial care, although ostensibly supportive, was paradoxically associated with lower nutritional adequacy in institutional contexts, underscoring the importance of autonomy in caregiving.

Socioeconomic and environmental factors, including urban residence and educational attainment, also influenced dietary patterns, pointing to the need for interventions that address both individual capacities and structural barriers. The multi-group invariance findings further suggest that etiology-specific pathways may require tailored approaches to nutrition and metabolic risk management.

Collectively, these findings support an integrated model of cardiometabolic risk in adults with disabilities, one that moves beyond traditional risk factor approaches to account for the complex interplay of functional, nutritional, psychosocial, and environmental determinants. Future longitudinal research is needed to establish temporal sequences and test whether interventions targeting nutritional adequacy, functional support, and mental health can modify cardiometabolic outcomes. From a clinical and policy perspective, these findings underscore the importance of integrated care models that address nutrition, physical function, mental health, and environmental barriers concurrently, rather than as disconnected domains. For adults with disabilities, cardiometabolic health is not merely a matter of diet or exercise alone, but of the systems of care, support, and environment that shape daily life.
